# Lower baseline testosterone level is related to earlier development of castration resistance in metastatic prostate cancer: a multi-center cohort study

**DOI:** 10.3389/fonc.2024.1321522

**Published:** 2024-02-20

**Authors:** Ho Ming Chris Wong, Peter Ka-Fung Chiu, Ignacio Puche-Sanz, Zhao Xue, Dong-Ning Chen, Enrique Gomez-Gomez, Isabel Heidegger, Mona Kafka, Yong Wei, Shinichi Sakamoto, Anthony Chi Fai Ng

**Affiliations:** ^1^ SH Ho Urology Centre, Department of Surgery, The Chinese University of Hong Kong, Hong Kong, Hong Kong SAR, China; ^2^ Division of Urology, Department of Surgery, North District Hospital, Hong Kong, Hong Kong SAR, China; ^3^ Division of Urology, Department of Surgery, Prince of Wales Hospital, Hong Kong, Hong Kong SAR, China; ^4^ Department of Urology, Hospital Universitario Virgen de las Nieves, Granada, Spain; ^5^ Department of Urology, Chiba University, Chiba, Japan; ^6^ Department of Urology, The First Affiliated Hospital of Fujian Medical University, Fujian, China; ^7^ Department of Urology, Medizinische Universität Innsbruck, Innsbruck, Austria

**Keywords:** prostate cancer, MHSPC, testosterone, combination (combined) therapy, mCRPC

## Abstract

**Purpose:**

In the era of concurrent combination therapy in metastatic hormone sensitive prostate cancer, the impact of the testosterone level before initiating androgen deprivation therapy on treatment outcome is still uncertain. We aimed to investigate its effect on time-to-castration-resistance in a metastatic hormone sensitive prostate cancer cohort.

**Methods:**

This is a multi-center retrospective study of 5 databases from China, Japan, Austria and Spain including 258 metastatic hormone sensitive prostate cancer patients with androgen deprivation therapy initiated between 2002 and 2021. Baseline testosterone was divided into high and low groups using 12 nmol/L as cutoff level. Primary outcome was time-to-castration-resistance. Secondary outcomes were survival functions. Kaplan-Meier method was employed to evaluate the correlation between baseline testosterone and time-to-castration-resistance. Subgroup analysis was performed to elucidate the effect of upfront combination-therapy and metastatic volume.

**Results:**

Median age was 72 years. Median follow-up time was 31 months. Median pre-treatment prostate-specific-antigen level was 161 ng/mL. Majority of case were graded as International-Society-of-Urological-Pathology grade 5 (63.6%). 57.8% patients had high volume disease and 69.0% received upfront combination treatment. 44.6% of the cohort developed castration-resistance. The low testosterone group demonstrated shorter mean-time-to-castration-resistance (19.0 *vs* 22.4 months, p=0.031). The variance was more significant in patients without combination therapy (13.2 *vs* 26.3 months, p=0.015). Cancer-specific and overall survival were inferior in the low baseline testosterone level group without receiving combination therapy (p=0.001).

**Conclusions:**

Lower pre-treatment testosterone level is correlated to shorter time-to-castration resistance and worse survival in metastatic prostate cancer patients without upfront combination therapy. Those with low baseline testosterone should be encouraged to adopt combination therapy to delay progression.

## Introduction

Prostate cancer (CaP) is a leading cause of death and a major healthcare burden in the developed world (1). Despite advancements in screening tests, early diagnosis and prompt treatment of localized CaP, a substantial amount of patients are diagnosed with primary metastatic disease (2, 3).

Long term androgen deprivation therapy (ADT) has remained the backbone of treatment for metastatic hormone sensitive prostate cancer (mHSPC) since 1940s (4, 5). Suppression of testosterone to castrate level below < 0.7 mmol/L has been the key to delaying progression in advanced CaP (6). However, little is known whether baseline testosterone level prior to the initiation ADT has an impact on the oncological outcomes of mHSPC.

Limited evidence demonstrated a higher baseline testosterone level was associated with slower PSA progression in CRPC, most of which were established in times when ADT was the only treatment option in advanced CaP (7, 8). As there is increasing evidence supporting upfront application of novel androgen receptor agents or chemotherapy in combination with ADT in the treatment of mHSPC, a combination therapy represents the new state of the art (9, 10). However, there has not been any updated literature looking into the effect of baseline testosterone level in this regard.

Therefore, we aimed to investigate the effect of baseline testosterone on treatment outcomes in terms of time to development of castration resistant prostate cancer (CRPC), based on this multi-center cohorts of mHSPC patients.

## Materials and methods

This is a retrospective analysis of data from 5 databases from China (Hong Kong and Fujian), Japan, Austria and Spain. We included patients with mHSPC at diagnosis. Only patients with documented serum testosterone level prior to any cancer treatment were included. All included patients had documented castrate level of testosterone <0.7nmol/L. Conventional imaging including bone scan and computer tomography (CT) scan, and next generation imaging namely PSMA PET-CT were accepted for the quantification of metastatic burden. Use of combination treatment was defined by the initiation of either chemotherapy or novel androgen receptor agents at the beginning of ADT treatment. The use of non-steroidal antiandrogens were not included in the analysis.

Baseline demographics, disease status, treatment strategies and oncological outcomes were documented. For baseline serum testosterone level, we adopted a cutoff of 12 nmol/L. Cases with ≥12nmol/L were classified as high-level group, while the rest were classified as low-level group (11). The primary outcome of interest is time to development of CRPC. It was defined as PSA progression of >25% above the nadir, confirmed by a second value 3 or more weeks later and an increase in absolute value of ≥2 ng/mL above nadir at least 12 weeks from baseline. Statistical comparison was carried out to evaluate the correlation between baseline serum testosterone level and time to CRPC. The secondary outcomes were survival functions including overall and cancer-specific survival.

Subgroup analysis was performed to elucidate the effect of metastatic volume and upfront combination therapy on the oncological outcome. For the classification of metastatic volume in our study, the definition of CHAARTED was used (10). High-volume metastatic disease was defined as the presence of visceral metastasis or ≥4 bone lesions with ≥1 beyond the vertebral bodies and pelvis.

Continuous variables are compared using Student’s t-tests. Categorical variables are compared using Fisher exact or chi-square tests as appropriate. Prognosis and outcomes were further analyzed by Kaplan Meier survival plots.

## Results

After collecting data from 5 multi-centre databases, a total of 258 mHSPC patients with initiation of ADT between 2002 and 2021 and documented serum testosterone level prior to treatment fulfilled the inclusion criteria and were analyzed.

Across the cohorts, the median patient age at diagnosis was 72 years (interquartile range [IQR]: 67 – 77). The median follow-up time was 31 months (IQR: 14.8 – 53). The median PSA level prior to ADT initiation was 161 ng/mL (IQR: 49.9 – 478.0). There were 184 patients (71.3%) with histological diagnosis of CaP. The International Society of Urological Pathology (ISUP) grades being 1-3, 4 and 5 were 8.7%, 27.7% and 63.6% respectively. The remaining patients were diagnosed and treated based on serum PSA and imaging findings.

There were 87 cases in the low testosterone group and 171 patients in the high testosterone group. The baseline patient characteristics were shown in **Table 1**. Comparing the two groups, most of the parameters did not show significant difference. The median PSA levels and ISUP gradings were similar. The mean number of bone metastasis and the number of cases with extra-vertebral bone or visceral metastases were also comparable.

**Table 1A:** Characteristics of patients stratified to low and high baseline testosterone groups.

	Low testosterone (<12nmol/L)	High testosterone (>=12nmol/L)	P value
Total number of patients	87	171	/
Median age at diagnosis (+- SD) years	73.1+-10.0	71.2+-7.9	0.135
Co morbidities			
• Diabetes mellitus	26.2%	21.8%	0.445
• Hyperlipidemia	15.5%	14.1%	0.777
Median PSA prior ADT (+- SEM) ng/ml	151.5 +-224	160.7 +- 97.1	0.356
ISUP grade group			0.207
• 1-3	3	13	
• 4	12	39	
• 5	33	66	
High volume metastasis	56.0%	59.0%	0.585
Visceral metastasis	29.0%	17.0%	0.077
Mean number of bone metastasis	14.7	11.7	0.356
Presence of extra-vertebral bone metastasis	69.0%	66.0%	0.750
Combination therapy	57.5%	74.9%	0.004

/, meaning P value is not applicable to this row.

**Table 1B:** Characteristics of patients with or without concurrent therapy.

	Concurrent therapy	No concurrent therapy	Total number/ p-value
Total number of patients	**178**	**80**	
Subgroup			
• High volume metastasis	134	15	N=149
• Low volume metastasis	44	65	N=109
• High testosterone (>=12nmol/L)	128	43	N=171
• Low testosterone (<12nmol/L)	50	37	N=87
Mean age (+-SD)	70.7+-8.6	74.6+-8.4	0.001
Median PSA prior ADT (+-SEM)	134.5+-126	180.0+-141	0.086
ISUP group			0.354
• 1-3	13	3	
• 4	48	3	
• 5	99	18	
Presence of visceral metastasis	20.3%	25.0%	0.627
Mean number of bone metastasis (+-SEM)	13.9+-1.5	4.5+-1.1	<0.001
Presence of co morbidities			
• Diabetes mellitus	25.6%	17.9%	0.186
• Hyperlipidemia	10.8%	23.1%	0.023

SD, standard deviation; PSA, prostate-specific antigen; SEM, Standard error of mean; ISUP, International Society of Urological Pathology.

178 patients (69.0%) received upfront combination therapy with either chemotherapy or novel androgen receptor agents. There were 149 patients (57.8%) with high-volume metastatic disease at diagnosis. Out of this group, 89.9% received upfront combination treatment. For patients with low-volume metastasis, only 40.4% of patients received combination therapy. The comparative characteristics of patients with or without combination therapy were depicted in **Table 1B**.

At the time of data cutoff, 115 patients (45.8%) developed CRPC. Among men who developed CRPC, 43 and 72 men were from the low and high testosterone group, respectively. Comparative mean times to CRPC were demonstrated in **Table 2**.

**Table 2:** Mean time to CRPC (in months) for patients with or without concurrent therapy.

	Low testosterone (n)	High testosterone (n)	P value
**Entire cohort (+-SEM)**	19.0 +- 4.1 (43 out of 87 cases developed CRPC)	22.4 +- 2.8 (72/171)	0.031
**Concurrent therapy**	23.3 +- 6.8 (25/50)	21.1 +- 3.4 (53/128)	0.18
**Without concurrent therapy**	13.2 +- 1.8 (18/37)	26.3 +-4.4 (19/43)	0.015
**p-value**	0.164	0.353	

SEM, Standard error of mean.

**Figure 1:**
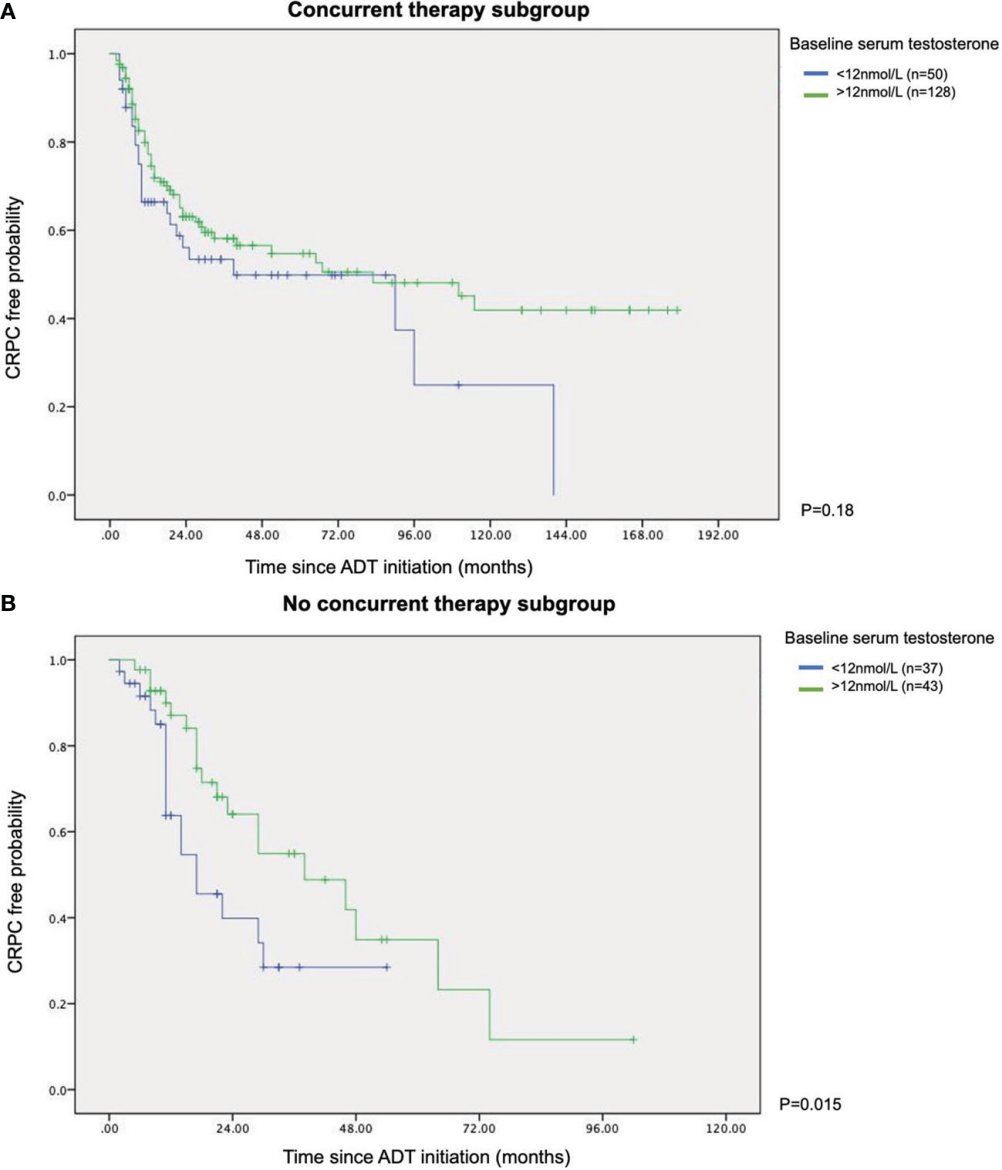
Kaplan Meier survival curves of with or without concurrent therapy in terms of time to CRPC **(A)** With concurrent therapy subgroup. **(B)** Without concurrent therapy subgroup.

In patients who did not receive combination therapy, lower testosterone was related to a much shorter mean time to CRPC than the high testosterone counterpart (13.2 *vs* 26.3 months, p=0.015). However, in men who had upfront combination therapy, the time to CRPC was not altered by baseline testosterone level. The results were depicted in the Kaplan Meier survival curves (see **Figure 1**).

**Figure 2:**
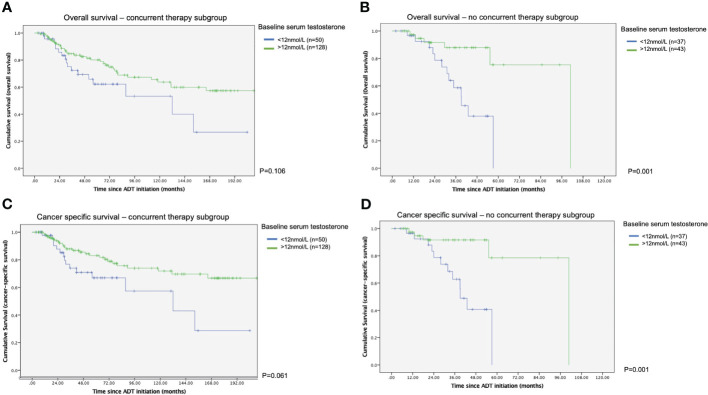
Kaplan Meier survival curves of with or without concurrent therapy in terms of time to survival **(A)** Overall survival – with concurrent therapy subgroup. **(B)** Overall survival - without concurrent therapy subgroup. **(C)** Cancer specific survival – with concurrent therapy subgroup. **(D)** Cancer specific survival - without concurrent therapy subgroup.

Survival outcomes were also inferior in patients with lower testosterone level prior to ADT. In the subgroup with no combination therapy, both cancer specific survival and overall survival were worse in those with baseline testosterone less than 12nmol/L (p=0.001 in both analyses), as represented in the Kaplan-Meier survival plots. In those who were treated with upfront combination therapy, the difference in survival functions did not reach a statistical significance (see **Figure 2**).

**Figure 3:**
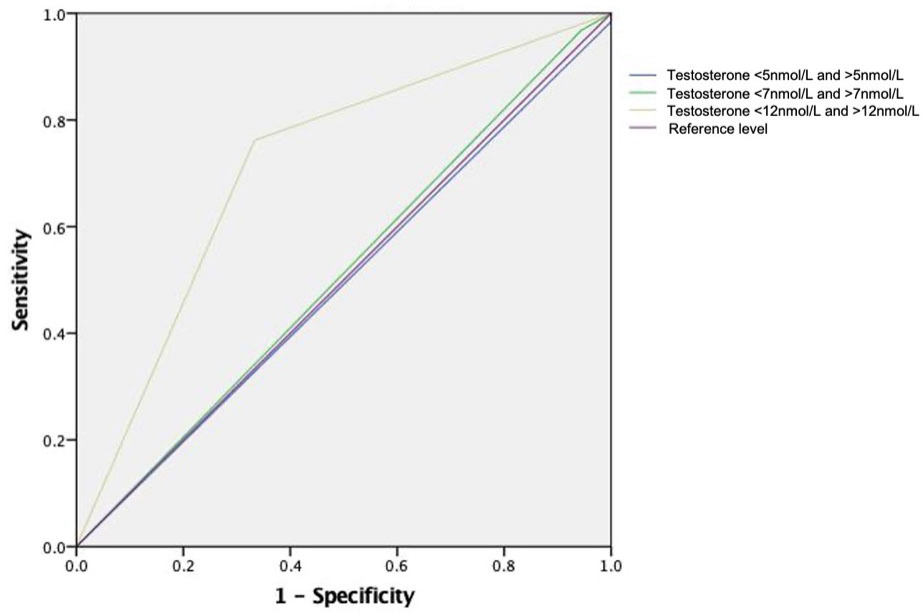
Receiver Operating Characteristic Curve for different testosterone cutoff level in predicting CRPC in 12 months time. Receiver operating characteristic curves for the models: using testosterone level of 12nmol as cutoff (area under ROC curve [AUC] 0.714), using 7nmol as cutoff (AUC 0.512) and using 5nmol as cutoff (AUC 0.492).

We further evaluated the performance of different testosterone cutoff levels in predicting time to CRPC in this mHSPC cohort. We narrowed down to cases with 1) time from ADT initiation to latest follow up ≥12 months and 2) from the low volume metastasis subgroup. Using 12nmol/L as the cutoff yielded an area-under-curve (AUC) of 0.714 in predicting the likelihood of CRPC in 12 months. Meanwhile, the AUC of using 7nmol/L and 5nmol/L were only 0.512 and 0.492 respectively (see **Figure 3**).

## Discussion

Testosterone level had been demonstrated to be associated with PSA expression, Gleason score, and androgen receptor (AR) expression in different stages of CaP, and hence disease progression (8). This has formed the scientific basis of ADT for treating advanced CaP. However, as time progresses, advanced CaP still evades castration and progresses to CRPC. Multiple postulations were suggested. Amplification of androgen receptor (AR) genes leads to hypersensitivity to ligand despite the lack of testosterone. Mutation to AR genes allow its activation despite a low testosterone seroenvironment. These were some of the theories that postulated tumor evolution despite an absence of testosterone (12). Here we hypothesize that for mHSPC presented itself in low baseline testosterone, it signifies the upregulation of testosterone-independent tumor growth pathways, which resulted in worse tumor control and shorter time to castration resistance. In the current study, mHSPC patients with low baseline testosterone were shown to benefit more from upfront combination therapy.

There were limited studies that investigated the effect of baseline testosterone level on the progression of advanced CaP. In a phase II-III trial of 101 mCRPC patients receiving salvage chemotherapy, de Liano and colleagues reported that among a group of castrated patients, those with testosterone level above median level of 11.5ng/dL had a better overall survival (OS) (10 months difference) and disease-free survival (DFS) (0.8 months difference) (13). Claps and colleagues analyzed 4 studies in their meta-analysis on baseline testosterone level and long-term advanced CaP outcomes. They described in a pooled analysis a significant association between baseline testosterone level and oncological outcomes including progression free survival (PFS) and OS (14–17).

It should be noted that currently available literature was based on dated studies, when the standard of care of advanced CaP was ADT monotherapy. Novel androgen receptor agents were limited to clinical trial usage. Our study is the first to describe the implication of baseline testosterone in the era of upfront combination therapy.

Here we adopted a dichotomous testosterone cutoff of 12nmol/L to classify patients to low and high baseline testosterone level. There has been no universally recognized baseline testosterone level that was adopted in CaP risk stratification models. One reference was made to a retrospective cohort of 762 Caucasian patients reported by Tu and colleagues (11). In their study, the subgroup of patients with testosterone <350ng/dL (equivalent to 12nmol/L) was associated with tumor aggressiveness and increased CaP-related mortality. It is paramount to also address that there has been no universally accepted way to classify or define normal baseline testosterone level. One related literature was a study that attempted to look into the effect of baseline testosterone level on clinically localized high risk prostate cancer due for radio therapy (18). Eastham and colleagues evaluated three different ways of characterizing testosterone level. They included a dichotomous cutoffs, as a continuous variable, and divided into quarters. It was found that there was no significant results demonstrated. Therefore, how the classification of low or high testosterone level and a normal cutoff were practically not present. We believe that the dichotomous cutoff of 12 nmol/L adopted in our analysis was reasonable.

In this study, we assessed whether baseline testosterone level would play a role in guiding the decision of early treatment intensification in the management of mHSPC. The argument comes in two folds. Firstly, despite the growing evidence body of combination therapy (9, 10), the real-world adoption of combination therapy at the instance of diagnosis was still suboptimal (18, 19), especially in the cases of limited metastasis. Our conclusion of baseline testosterone potentially playing a role in the oncological outcomes may offer yet one more reason for physicians to encourage the use of combination treatment in the low-volume-metastasis and low-testosterone-level subgroup. To be more specific, there would be potential benefits of adopting a dichotomous cutoff in clinical usability. Clinicians could potentially take reference to such a marker easily in a clinical decision process. Potentially testosterone level can be checked in all newly diagnosed mHSPC patients. For those that were found to have low volume metastasis and that were fit for but were not motivated to receive combination treatment due to various reasons can be especially counselled. Clinicians could encourage the adoption of combination therapy to those identified with a low baseline testosterone. Secondly, the exploration of the baseline testosterone level as a negative prognostic predictor could bring implication to further investigation. In an era when multiple frontiers are exploring evidences to support alternative strategies in the management of low-volume mHSPC, such as triplet therapy or external beam radiotherapy (19), it would be fascinating to see whether baseline testosterone level would serve a role in terms of personalized medicine.

From our results, the time to CRPC was not altered by the serum testosterone level in those that were treated with concurrent therapy, nor were the cumulative survival figures. Metastatic CaP that nonetheless developed in low testosterone may signify preexisting aberrant androgen receptor (AR)activity or earlier activation of testosterone independent pathway and hence presence of castration resistant clones. In an exploratory retrospective study (20), Lolli and colleagues investigated mCRPC patients treated with enzalutamide or abiraterone. They identified the relationship between lower baseline testosterone level and high AR copy number (which was one of the mechanisms of AR aberrant activity). They were found to be negative prognostic predictor of survival outcomes. This could potentially mean cancer clones in low testosterone environment achieved quicker evasion of castration, thus shorter time to CRPC. Castration independent pathways were inhibited by novel androgen receptor agents via AR signal pathway suppression, or by chemotherapy via direct antineoplastic activity. With both androgen dependent and independent pathways inhibited by castration and concurrent therapy, the time to CRPC was thus not altered by baseline testosterone level.

There were limitations in our current study that should be addressed. Being a retrospective review - despite the best effort - our analysis only demonstrated the predictive value of low testosterone level towards treatment efficacy of mHSPC. A causal relationship could not be established. Data was collected in multiple centers. This would imply variances in total serum testosterone assays adopted in different centers. While it was described that mean difference across testosterone assays amounted to around 5 to 10%, its effect should not be totally neglected (21). However, such intrinsic variation could not be eliminated by the current methodology. Moreover, testosterone level was multifactorial with confounders difficult to account for. Differences in general health, dietary pattern, disease progression and mobility status were some of the examples. This could lead to confounding bias to our case classification. Another potential limitation to our analysis would be the difficulty to account for all the treatment related confounders due to a rapid shift of the mHSPC treatment landscape. For one, with more recent evidences demonstrating relative survival benefit from radiotherapy combined with ADT or combination therapy in low volume mHSPC (19), the inability of the current analysis to account for this factor could potentially introduce bias to our secondary outcomes (survival figures). The same applies for the multitude of individual treatment options that emerged in the recent years. It would not be statistically efficient to segregate all the options and perform a multivariate analysis, given our relatively limited sample size despite all effort. Nonetheless, the authors would like to point out that with our attempt to maximize case inclusion for a more thorough analysis in a limited territory, it would be inevitable to include these historical cases. With the relatively similar baseline characteristics between the low and high baseline testosterone cohorts, we believe the above results still brought meaningful insights despite noticeable limitations.

On the other hand, there are merits of our publication that make it relevant for the discussion. We were able to establish a rather homogenous cohort that limits cases presented with metastatic disease. This aided to answer the clinical question of whether testosterone level affects the decision of offering concurrent therapy at first instance. Our study results were also based on real-world clinical scenario. Despite the promising result of upfront therapy in mHSPC, the real-world usage is still suboptimal (22, 23). Our set of data included patients that were presented with different degrees of metastasis, and who had been offered different types of concurrent therapy. It might provide some guidance as to which patient subgroup might particularly benefit from upfront therapy in terms of delaying progression to CRPC.

## Conclusion

Baseline testosterone level is a prognostic predictor of disease progression in terms of time to CRPC, which translated to implications in survival outcomes in mHSPC patients with no concurrent therapy. This subgroup of patients with low baseline testosterone may potentially be encouraged to receive concurrent therapy in addition to androgen deprivation. The analysis of baseline testosterone level should be included in men with mHSPC prior to treatment discussion.

## Data availability statement

The original contributions presented in the study are included in the article/supplementary material. Further inquiries can be directed to the corresponding author.

## Ethics statement

Ethics approval had been granted for the conduct of this study.

## Author contributions

HW: Conceptualization, Data curation, Formal analysis, Investigation, Validation, Writing – original draft, Writing – review & editing. PC: Conceptualization, Data curation, Investigation, Methodology, Project administration, Resources, Supervision, Writing – review & editing. IP: Data curation, Writing – review & editing. ZX: Data curation, Writing – review & editing. DC: Data curation, Writing – review & editing. EG: Data curation, Writing – review & editing. IH: Data curation, Writing – review & editing. MK: Data curation, Writing – review & editing. YW: Data curation, Writing – review & editing. SS: Data curation, Writing – review & editing. AN: Supervision, Writing – review & editing.
